# Cytomegalovirus anterior uveitis and occlusive retinal vasculitis without retinitis in a patient on immunomodulatory therapy

**DOI:** 10.1186/s12348-023-00356-z

**Published:** 2023-08-04

**Authors:** Lorenzo Fabozzi, Ilaria Testi, Laura De Benito-Llopis, Carlos Pavesio

**Affiliations:** 1 Department of Uveitis, Moorfields Eye Hospital, National Health Service Foundation Trust, London, UK; 2 Rheumatology Department, Great Ormond Street Hospital for Children, London, UK; 3 Department of External Diseases, Moorfields Eye Hospital, National Health Service Foundation Trust, London, UK

**Keywords:** Cytomegalovirus virus, Uveitis, Endotheliitis, Retinal vasculitis, Retinitis

## Abstract

**Purpose:**

To describe unusual clinical features and therapeutic management of a case of cytomegalovirus (CMV) ocular disease in a patient on immunomodulatory therapy.

**Setting/venue:**

Moorfields Eye Hospital NHS Foundation Trust, London, UK.

**Methods:**

Medical history, clinical findings, investigation results, and multimodal imaging were retrospectively collected.

**Results:**

A 61-year-old, South-East Asian man, developed CMV-related endotheliitis and occlusive retinal vasculitis, diagnosed by wide-angle fluorescein angiography. No retinitis was present on the fundus examination. Suspicion of CMV etiology was based on anterior segment findings, especially the presence of coin-shaped endothelial lesions. The diagnosis was confirmed by aqueous polymerase chain reaction (PCR) analysis which was positive for CMV DNA. The combined use of topical and systemic valganciclovir resulted in significant improvement of the picture.

**Conclusions:**

CMV can manifest in the eye as occlusive retinal vasculitis without the presence of typical retinitis.

## Introduction

Cytomegalovirus (CMV) is a ubiquitous, enveloped, and double-stranded DNA virus of the Herpesviridae family [1]. Primary CMV infection is typically mild or asymptomatic in immunocompetent individuals [2]. However, CMV infections can cause permanent damage in the setting of congenital infection and immunocompromised individuals. Acute anterior uveitis, corneal endotheliitis and retinitis have been associated with a CMV eye infection [1, 3]. We report an atypical case of CMV-related occlusive retinal vasculitis manifesting without retinitis in a non-severely immunocompromised patient on immunomodulatory therapy, associated with anterior uveitis and corneal endotheliitis, whose prompt diagnosis and treatment resulted in marked anatomical improvement and also functional improvement.

## Case presentation

**Fig. 1:**
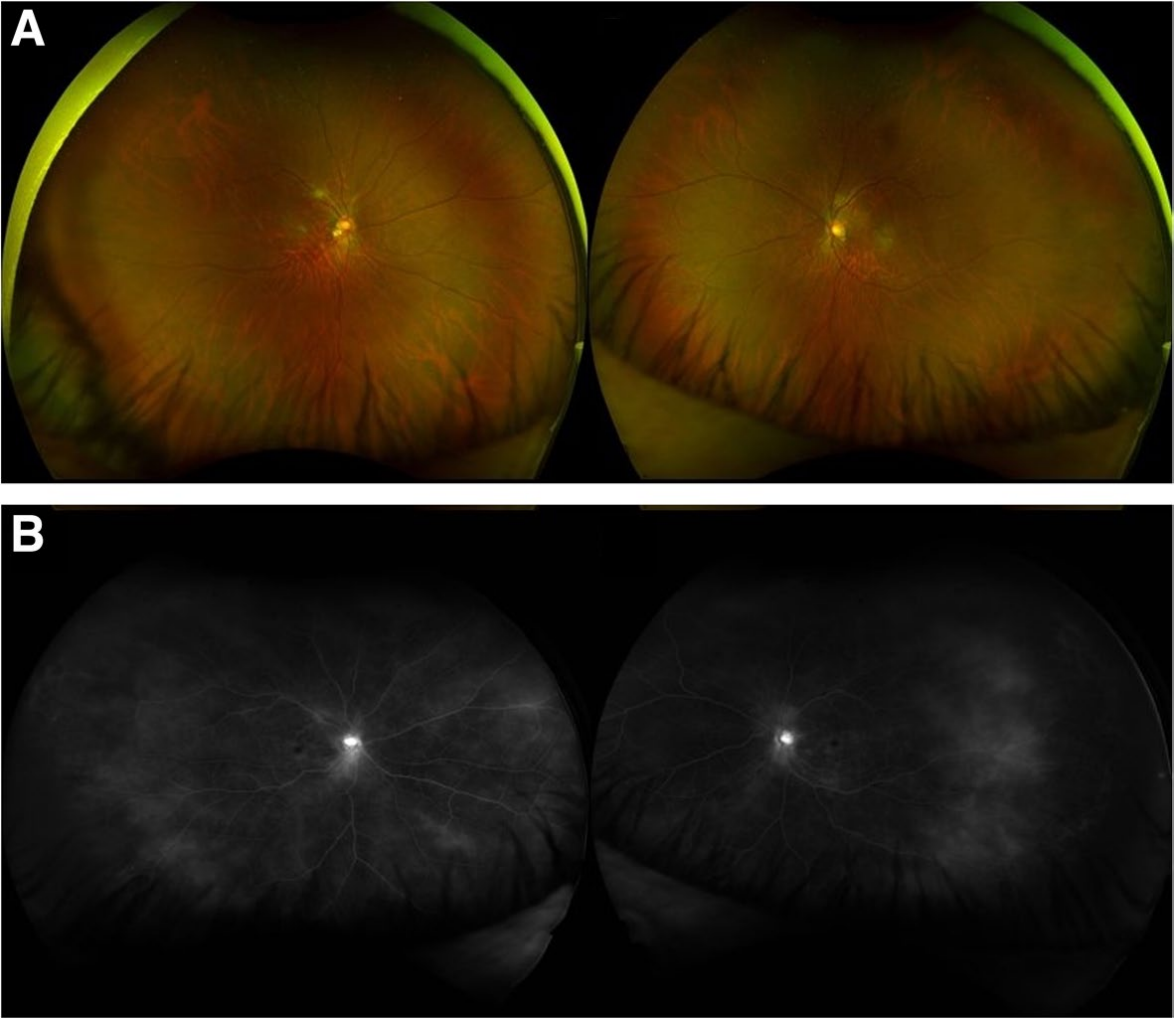
**A** Optos ultra-widefield pseudocolour fundus picture showing bilateral cotton wool spots, located along the temporal arcades in the right eye and along the avascular arcade surrounding the optic disc in the left eye. **B** Optos ultra-widefield fluorescein angiography showing bilateral vascular leakage in the absence of areas of capillary non-perfusion

A 61-year-old South-East Asian male presented to the Uveitis Service complaining of a decrease in vision in both eyes for a few weeks. His general health was unremarkable, and he denied any other symptoms/signs. The patient was high myopic with pseudophakia in both eyes and a past ocular history of diplopia related to decompensating left 4th nerve palsy, corrected with a Fresnel prism prescription. He underwent cataract surgery on the right eye in July and the left eye in September. One month after left eye surgery he reported that the vision in the right eye was not clear. On clinical examination, the best corrected visual acuity (BCVA) was 6/15 in both eyes. Intraocular pressure was 24 mmHg in the right eye and 23 mmHg in the left eye. Bilateral anterior segment examination revealed inferior keratic precipitates (KPs) and 0.5 + anterior chamber cells. On dilated fundoscopy, both eyes showed resolving cotton wool spots (CWS), located along the temporal arcades in the right eye and along the avascular arcade surrounding the optic disc in the left eye, in the absence of chorioretinal lesions (Fig. 1A). Vascular involvement was not clinically detectable. However, Optos ultra-widefield (UWF) fundus fluorescein angiography (FFA) showed bilateral vascular leakage and the absence of areas of capillary non-perfusion (Fig. 1B). The absence of choroidal involvement was confirmed by indocyanine green angiography (ICGA). Optical coherence tomography (OCT) showed a bilateral dry macula. The patient was diagnosed with bilateral anterior and intermediate uveitis. Full blood count, kidney and liver function, serum angiotensin-converting enzyme, C-reactive protein, erythrocyte sedimentation rate, antineutrophil cytoplasmic antibodies, antinuclear antibodies, anti-double stranded DNA antibodies, treponemal serology and Quantiferon TB Gold were all normal/negative. The patient was started on a tapering course of Prednisolone 0.8 mg/kg/day together with topical dexamethasone.

**Fig. 2:**
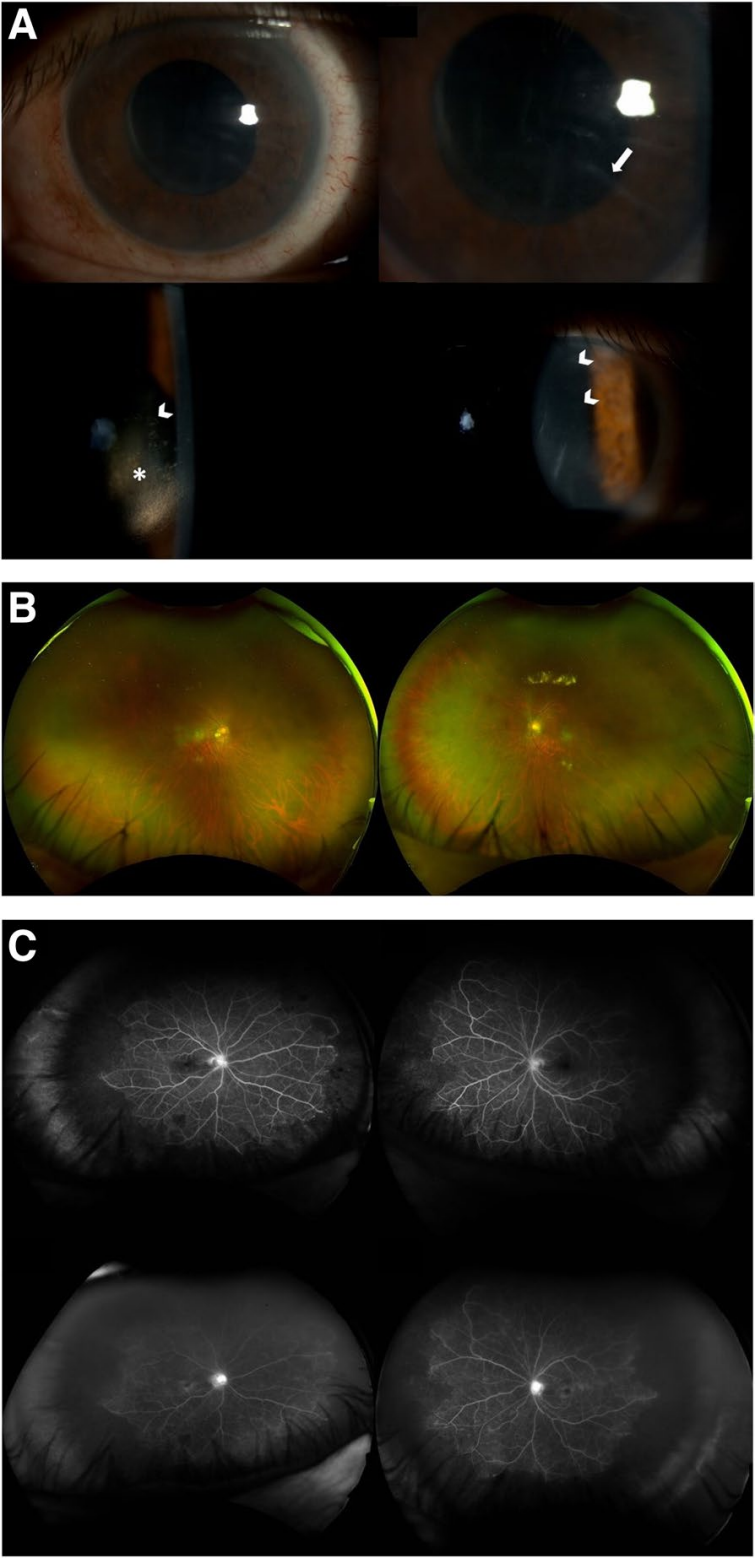
**A** Topcon anterior segment photographs of both eyes showing CMV endotheliitis with Descemet’s membrane folds (white arrow), corneal oedema (asterisk), and coin-shaped keratic precipitates (white arrowheads). **B** Optos ultra-widefield pseudocolour fundus picture showing bilateral peripheral retinal hemorrhages. **C** Optos ultra-widefield fluorescein angiography (early and late phases) showing bilateral severe occlusive vasculitis with peripheral areas of capillary non-perfusion

At the 8-week follow-up, when the patient was on Prednisolone 10 mg/day, the ophthalmic examination did not show signs of intraocular inflammation, and a repeat of FFA showed bilateral residual peripheral vascular leakage and left persistent capillary leakage at the macula. A second-line immunomodulator steroid-sparing agent (mycophenolate mofetil) was started. Four weeks later, the patient came back with a bilateral decrease in visual acuity and high intraocular pressure in the right eye. The IOP was 28 mmHg in the right eye and 16 mmHg in the left eye. Both eyes showed 0.5 + anterior chamber cells with small KPs and unremarkable posterior segment examination. Lowering intraocular pressure eye drops and topical dexamethasone were started. Four-week follow-up showed no inflammation and normal intraocular pressure in both eyes. Eight weeks later, on MMF 1 g bd for 12 weeks and Prednisolone 7.5 mg/day, the patient presented again with decreased vision in both eyes. Ophthalmic examination revealed bilateral mild anterior chamber inflammation and new onset of bilateral vitritis. Prednisolone was therefore increased up to 40 mg once a day, and a decision to start Adalimumab was made. However, four weeks later, while waiting for the Adalimumab to be started, the patient presented with a bilateral reduction in vision (BCVA was 6/24 in the right eye and 3/60 in the left eye), normal intraocular pressure, corneal oedema with Descemet’s membrane folds and coin-shaped endothelial lesions (Fig. 2A), mild anterior chamber inflammation, and peripheral retinal haemorrhages (Fig. 2B). OCT showed a dry macula in both eyes. Ultra-widefield FFA showed bilateral severe occlusive vasculitis with peripheral areas of capillary non-perfusion (Fig. 2C). Based on the characteristic anterior segment features, CMV endotheliitis was suspected and an anterior chamber tap was performed. Aqueous polymerase chain reaction (PCR) analysis was positive for CMV. MMF was stopped, Ganciclovir 0.15% eye gel 5 times a day and Valganciclovir 900 mg bd were started, with topical and oral steroids continued. Two weeks later there was an improvement of the endotheliitis with near complete resolution of the coin-shaped lesions. Corneal oedema had improved but was not completely resolved. Repeated ultra-widefield FFA at 8 weeks showed significant peripheral ischemia in both eyes, requiring laser panretinal photocoagulation. The patient continued on a maintenance dose of Valganciclovir tablets 450 mg twice a day. 6 weeks later, his right cornea was almost completely clear, but the left still showed inferior oedema. BCVA was 6/12 in the right eye and 6/24 in the left eye. The first laser panretinal photocoagulation session was performed in both eyes.

## Discussion

We reported a case of CMV occlusive retinal vasculitis manifesting without the characteristic retinitis in a patient receiving immunomodulatory therapy for an initial picture of bilateral retinal vasculitis, showing the typical features of CMV endotheliitis. Several clues may increase the suspicion of CMV anterior uveitis, including high IOP, low-grade anterior chamber inflammation, coin-shaped corneal lesions and small keratic precipitates [4, 5]. A suggestive clinical phenotype and the detection of CMV DNA from aqueous samples are required to achieve a confirmed diagnosis of CMV anterior uveitis [6].

CMV retinal involvement commonly occurs in severely immunocompromised patients, including subjects with acquired immune deficiency syndrome (AIDS), and those undergoing chemotherapy and organ transplants [7]. Typically, the areas of CMV retinitis are located along the retinal vessels, so their role in the pathogenesis of retinitis has been hypothesized. In addition, immunohistochemical studies detected CMV proteins in the retinal vascular endothelium adjacent to the areas of retinal involvement, with the vascular endothelium being proposed as the primary site of CMV infection in the retina [8–10]. Involvement of retinal vessels, both arteries and veins, is common in CMV retinitis. In HIV patients, the exact role of HIV vasculopathy in the development of CMV retinitis is still controversial [7, 11]. Patients with HIV infection and CMV retinitis have a predominant involvement of retinal veins, consisting of perivenous sheathing [12, 13]. Pathanapitoon et al. reported a high prevalence of vascular involvement in their retrospective observational case series of 22 eyes with CMV associated posterior uveitis without HIV infection [11]. Eleven of 18 patients were taking immunosuppressive medications, 1 had non-Hodgkin lymphoma, 1 had primary immunodeficiency disorder, 2 had diabetes mellitus, and 3 had no systemic diseases and no evidence of immune deficiency. The authors reported 13 eyes with focal hemorrhagic retinitis, 7 with peripheral retinal necrosis and 2 with vasculitis and vitritis without focal retinal lesions. Retinal vasculitis was observed in 16 of 22 eyes, and a primary involvement of retinal arteries was observed (13 of 16 eyes) [11]. Another presentation of CMV vasculitis is frosted branch angiitis, which can be associated with multiple infective agents [14].

No cases of CMV-related occlusive vasculitis in the absence of retinitis have been reported so far in the literature. Our patient initially had no obvious clinical features to suspect a CMV infection and was diagnosed with idiopathic anterior and intermediate uveitis. Different theories can be hypothesized, including an acquired infection/reactivation of CMV infection, leading to the characteristic CMV endotheliitis and a change in the features of the retinal vasculitis from non-occlusive to occlusive vasculitis. The role of mycophenolate mofetil in the appearance of the CMV infection/reactivation is controversial, considering that was used as an immunomodulatory agent in a patient who was not profoundly immunosuppressed.

## Conclusion

CMV infection can result in occlusive vasculitis in the absence of retinitis, but it is uncertain if this can happen as a primary insult or will happen in cases of ongoing vasculitis (double hit).

## Data Availability

All data and images pertaining to the cases are available with the corresponding author.
